# Single-incision approach to aesthetic flat closure after bilateral mastectomy in morbidly obese patients

**DOI:** 10.1016/j.jpra.2023.10.014

**Published:** 2023-11-03

**Authors:** Jean-Claude D. Schwartz

**Affiliations:** Northside Hospital, Department of Surgery Gwinnett Surgical Specialists 631 Professional Drive Suite 300 Lawrenceville, GA 30046, USA

**Keywords:** Aesthetic flat closure, Going flat, Oncoplasty, Mastectomy

## Abstract

There has been a recent emphasis to offer women who forego reconstruction after mastectomy a surgically optimized flat closure (aesthetic flat closure (AFC)). While this certainly requires attention to details not previously considered by many surgeons, additional complexity is encountered in performing this procedure in the morbidly obese patient. Most of this additional complexity revolves around resecting additional subcutaneous tissue adjacent to the breast footprint. Here, we combine two previously described techniques, one to facilitate AFC in patients with normal body mass indices (BMIs) and another approach used to facilitate removal of excess lateral subcutaneous tissue after mastectomy in patients with elevated BMIs with our single-incision approach. The single-incision approach facilitates an expedited surgical procedure and resection of excess midline tissue with more reliably symmetrical incisions bilaterally . This report describes 10 consecutive morbidly obese patients who underwent mastectomy and AFC.

## Introduction

Obese patients are at higher risk for developing breast cancer,^1^^,^^2^ many of whom undergo mastectomy. Many forgo reconstruction given their higher rates of reconstructive failure^3^ and demand AFC to leave them as flat as possible.^4^ The National Cancer Institute defines AFC as a surgery after one or both breasts are removed where extra skin and fat in the breast area is removed, tightening the remaining tissue so that the chest wall appears flat.^5^

Karp et al.^6^ described basic principles for AFC which include obliteration of the inframammary fold (IMF), resection of excess skin and medial and lateral fatty tissue with careful attention to symmetric incisions and equivalent thickness between superior and inferior mastectomy flaps. However, excision of excess lateral tissue in the morbidly obese is more complex and requires additional considerations. While there have been numerous strategies described for this,^7^ the author favors the approach described by Clough where excess lateral skin and fat are excised through a vertical incision with subsequent medial advancement and thinning out of the lateral skin flap overlying the latissimus dorsi.^8^^,^^9^ In addition to adopting the approaches by Karp and Clough, a single large incision is used which further expedites bilateral mastectomy and AFC. This report describes ten consecutive morbidly obese patients where this modified approach is utilized.

## Patients and methods

**Figure 1:**
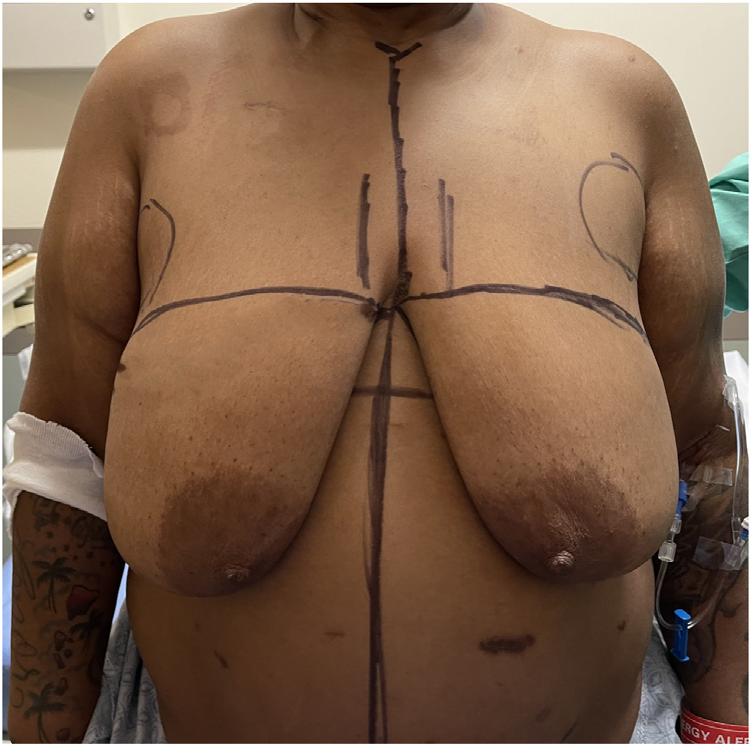
48 year-old female with a BMI of 37 kg/m^2^ with left breast cancer. She has a history of gastric bypass. Her superior mastectomy incisions are marked as are the fatty pre axillary areas which require debulking to obtain a flat closure. Her inferior mastectomy incisions are placed in the inframammary fold.

**Figure 2:**
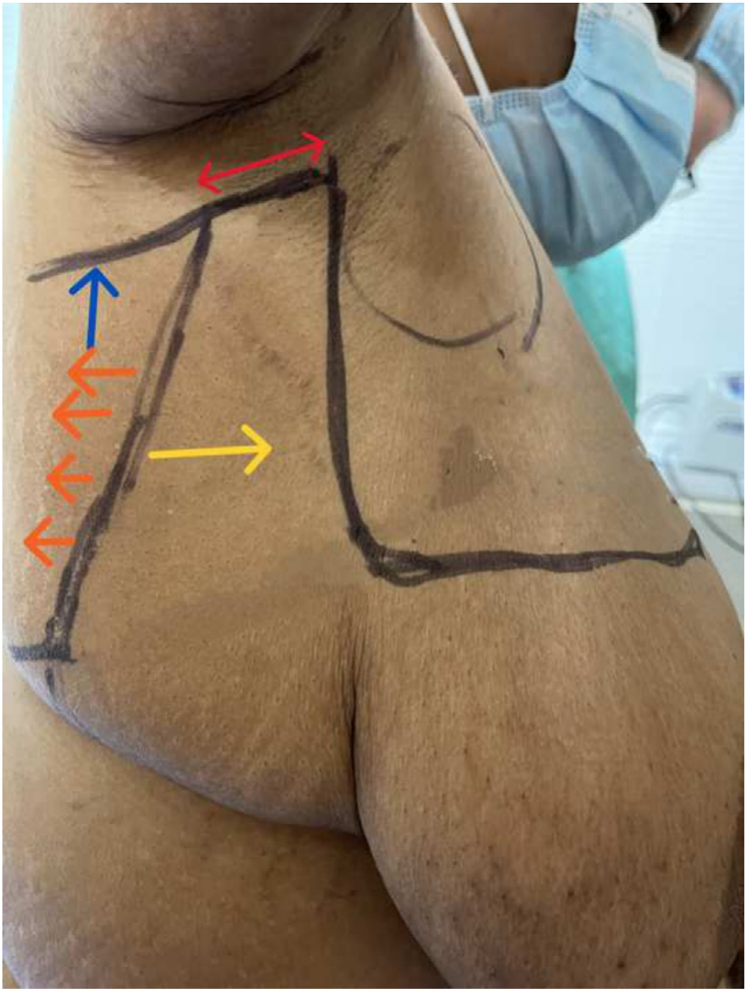
This is a lateral view, right side demonstrating our preoperative markings. The skin between the two vertical parallel lines is excised along with the underlying fat. This vertical excision reduced the horizontal excess in the axilla, facilitating a flat lateral contour. In reality, often more skin and fat is excised, after tailor-tacking during closure. The bi-directional red arrow signifies the area of skin that will be completely excised facilitating medial advancement (yellow arrow) of the thinned out (orange arrows) lateral skin flap overlying the latissimus muscle. The blue arrow represents the posterior back cut which facilitates medial advancement of the skin flap.

**Figure 3:**
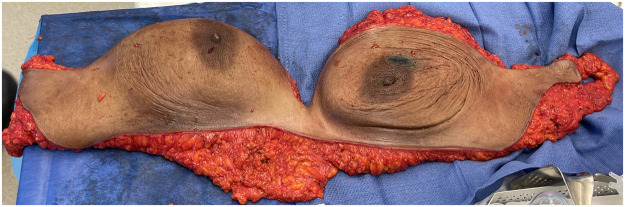
This demonstrates the bilateral mastectomy specimen which is resected en bloc. The single-incision approach facilitates a quicker surgical procedure, better exposure, optimal midline debulking of tissues and more reliably symmetrical incisions.

**Figure 4:**
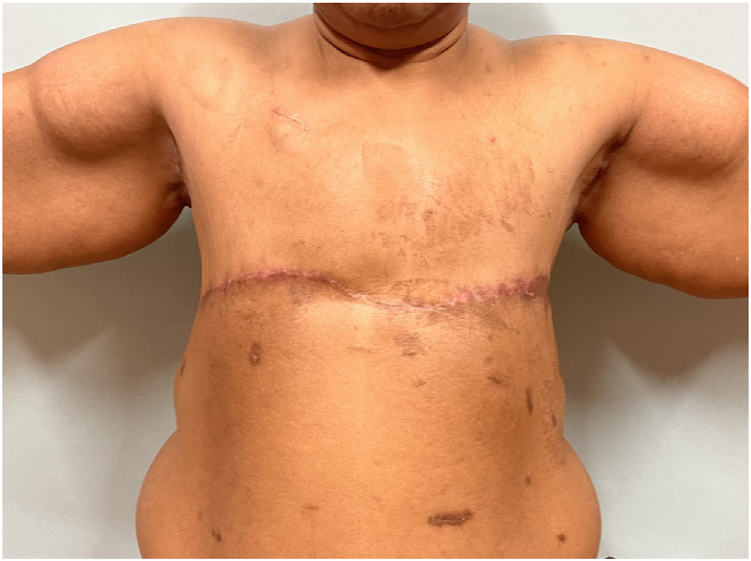
Four month post-operative picture demonstrating this patient's AFC. Her surgical procedure is documented in Supplemental Digital Content, Video 2. The most challenging portion of these procedures is insuring a flat lateral contour with no step off between the anterior mastectomy incision and the lateral chest wall.

Retrospective review of ten consecutive morbidly obese patients that underwent this surgery between February 2021 and January 2023 was performed. Patients were marked with a single transverse incision across both breasts, denoting the superior mastectomy incision (Figure 1; Supplemental Digital Content, Video 1). The inferior incision was placed in the IMF, spanning both breasts. The upper incision was then curved superiorly at the anterior axillary line to the axillary crease (Figure 2). Similarly, the IMF incision was curved superiorly at the posterior axillary line to the axillary crease, leaving ∼ 3–4 cm of intervening skin between these two parallel lines, which was excised. A back cut starting at the posterior axillary line in the axillary crease was then performed which allowed for medial advancement after the flap was defatted (Figure 2). The bilateral mastectomy was then performed (Supplemental Digital Content, Video 2). The IMF was then obliterated and undermined ∼ 10 cm to facilitate tissue advancement superiorly. Similarly, the superior flap was mobilized until the clavicle was palpable to facilitate closure. While skin and fat was excised in the midline en bloc with the left and right mastectomies (Figure 3), less aggressive skin resection was performed as there is less excess skin here. Both mastectomy specimens were removed in continuity along with the excess lateral fatty tissues overlying both LD muscles. Three round Blake drains were placed on each side. The mastectomy flaps were quilted with multiple rows of 2–0 absorbable suture. The incisions were then closed (Figure 4) and a light wrap was applied. Unilateral mastectomy surgery was performed in a similar fashion with the incision made just across midline to facilitate excision of the medial dogear.

## Results

Patient age and body mass index ranged from 35 to 78 years (mean = 53.4, SD = 13.5) and 35.1 to 46.2 kg/m2 (mean = 39.8, SD = 3.9), respectively. All patients underwent bilateral mastectomy and AFC with at least 6 months follow-up. Operative times ranged from 89 to 157 min (mean = 125.1 min, SD = 19.2 min). Mastectomy weights ranged from 752 to 2136 gs (mean = 1215.3 gs, SD= 382.9 gs). Four patients (40 %) developed wound healing complications (three underwent previous radiotherapy) and were successfully treated with outpatient wound care within 8 weeks. Two patients (10 %) developed persistent seromas requiring return to surgery for drainage. There were no post-operative hematomas requiring reoperation.

## Discussion

Many mastectomy patients choose to forego reconstruction.^4^ Instead, they “go flat” which requires that surgeons become proficient in techniques that leave patients with no residual evidence of the breast footprint. Karp et al.^5^ described an effective approach to achieve this in patients with normal range BMIs. These patients don't have vast amounts of tissue adjacent to the breast footprint and minimal additional work is required to debulk these areas. However, in morbidly obese patients, there is significant lateral adiposity that must be excised to minimize the step-off between the mastectomy and lateral chest wall to optimize the aesthetic outcome. This excess lateral fullness must be addressed in both vertical and horizontal dimensions to minimize the residual dogear. In the author's opinion, this excess lateral tissue is best excised using a technique described by Clough et al.^8^^,^^9^ with a “J” (left breast) or “L” (right breast) incision where an additional vertical scar is present in the mid-axillary line which allows for aggressive excision of any lateral horizontal excess. This also allows for medial advancement and defatting of the skin flap overlying the latissimus which leaves the patient with a flat lateral chest wall contour with no significant step-off with the anterior mastectomy incision (Supplemental Digital Content, Video 2 and Figures 2 and 4).

In addition to synthesizing the approaches described by Clough and Karp, bilateral surgeries are performed through one incision. This expedites the surgery, providing optimal exposure, as both mastectomies are performed simultaneously. This also allows for direct excision of excess midline subcutaneous tissue which can be considerable in obese patients. These midline tissues, if left behind, can result in a significant step-off with the mastectomy cavity resulting in a poor aesthetic outcome. The single-incision approach also allows for more reliably symmetric incisions on both sides. Caution should be exercised in patients with a history of radiotherapy as the resultant mastectomy flaps are more ischaemic and less mobile making mobilization and closure more challenging.

The surgical approach described here is more complex and requires longer operative times than would a traditional mastectomy with linear closure. In patients who are already at higher risk of operative complications, one must weigh these additional risks with the obvious aesthetic benefits and functional consequences of leaving excess lateral tissues behind (chafing, sensation of fullness/heaviness, interference with arm movement and difficulty fitting a brassiere/clothing).

## Conclusions

AFC in morbidly obese patients requires a different strategy for successful outcome. By combining previously described approaches for AFC in patients with normal range BMIs and another technique for excision of excess lateral adiposity in the obese after mastectomy with the single-incision approach presented here, reliably good outcomes can be obtained for these challenging patients.

## Conflicts of interest

None declared.

## Appendix Supplementary materials

Image, application 1

Image, video 2

Image, video 3
