# Supplementary material for: Single-incision approach to aesthetic flat closure after bilateral mastectomy in morbidly obese patients
Source: JPRAS Open. 2023 Nov 3;39:18–22. doi: 10.1016/j.jpra.2023.10.014 (PMC10724483; doi:10.1016/j.jpra.2023.10.014)
Supplement: Supplementary file 1 [file mmc1.docx]

Supplemental Digital Content

Video 1. Preoperative markings for aesthetic flat closure in a morbidly obese patient.

Video 2. This is a video demonstrating the key steps in an aesthetic flat closure in a morbidly obese cancer patient undergoing bilateral mastectomy.
